# The Impact of Natural Deep Eutectic Solvents and Extraction Method on the Co-Extraction of Trace Metals from *Fucus vesiculosus*

**DOI:** 10.3390/md20050324

**Published:** 2022-05-13

**Authors:** Alexander N. Shikov, Ekaterina D. Obluchinskaya, Elena V. Flisyuk, Inna I. Terninko, Yulia E. Generalova, Olga N. Pozharitskaya

**Affiliations:** 1 Murmansk Marine Biological Institute of the Russian Academy of Sciences (MMBI RAS), Vladimirskaya, 17, 183010 Murmansk, Russia; obluchinskaya@gmail.com (E.D.O.); olgapozhar@mail.ru (O.N.P.); 2 Department of Pharmaceutical Formulations, St. Petersburg State Chemical Pharmaceutical University, Prof. Popov, 14, 197376 Saint-Petersburg, Russia; elena.flisyuk@pharminnotech.com; 3 Core Shared Research Facilities “Analytical Center”, St. Petersburg State Chemical Pharmaceutical University, Prof. Popov, 14, 197376 Saint-Petersburg, Russia; inna.terninko@pharminnotech.com (I.I.T.); generalova.yuliya@pharminnotech.com (Y.E.G.)

**Keywords:** natural deep eutectic solvents, *Fucus vesiculosus*, trace elements, trace metals, extraction, hazard quotient, health risk, NADES

## Abstract

In recent years, natural deep eutectic solvents (NADES) have been widely investigated for the extraction of food and medicinal plants as well as seaweeds. However, the ability of NADES for trace elements co-extraction from natural sources is not well investigated. The aim of this study was to investigate the ability of common NADES for trace elements co-extraction from *Fucus vesiculosus*. All of the tested NADES did not recover As and Co (concentration <LOQ). Moreover, all of the tested NADES provided a low recovery (<9%) of Ba, Ca, Fe, Mg, Mn, Sr, and Zn. The method of extraction had not shown a statistically significant effect on the co-extraction of all elements (excluding Ba and Ca). In contrast, the water content in NADES was significantly affected on the recovery of Ba, Ca, Mg, Mn, Sr, and Zn. The recovery of Al and Cr was relatively high and considerably varied (from 1.5 to 59.9%). NADES comprising lactic acid:glucose:H_2_O (5:3:1) provided the lowest contents of all elements, and the highest extracted amounts were obtained employing water contents of 60–80%. The calculated daily intake of all the elements contained in NADES extracts were less than the daily dose risk estimators. The hazard quotients, hazard indexes, and carcinogenic risk calculated for all trace elements and their combination were considerably less than 1. This evidences no health risk, and carcinogenic risk after topical application of all studied NADES. For the first time, the results of the current study demonstrated that NADES extracts of *F. vesiculosus* contain a lower amount of trace metals and are safer than the extracts obtained with water and 70% acetone. This indicates a significant advantage for NADES compared with the other solvents.

## 1. Introduction

The natural deep eutectic solvents (NADES) are considered as a new class of green solvents that are useful for the extraction of different pharmacologically active compounds from natural sources. Basically, to be polar solvents, NADES are described for the extraction of phenolics [1,2], anthocyanins [3,4], and flavonoids [5,6]. Furthermore, these solvents were adjusted for compounds with lower polarity, such as aglycons of phenyletanes and phenylpropanoids [7], astaxanthin [8], fucoxanthin [9], steroidal saponins [10], iridoids [11,12], anthraquinones [13], etc.

Osowska and Ruzik (2019) proposed a series of NADES for the extraction of Mn, Co, Cu, Zn, and Mo from young barley [14]. NADES with the water content of 10% were reported as effective solvents for the removal of Pb, Cd, Cr, As, and Cu from the red algae *Porphyra haitanens* [15]. The pollution of phytoextracts with trace elements is one of the crucial problems. Trace elements could significantly affect the safety and stability of formulations and cause side effects. The authors of the above-mentioned publications have tuned solvents for the selective extraction of trace elements. However, little is known regarding the ability of NADES for trace elements co-extraction. 

The brown seaweed *Fucus vesiculosus* attracts the attention of specialists as a rich source of polysaccharides, phlorotannins, carotenoids, and other active compounds, which shows potent activities as antioxidants [16], antiangiogenic [17], anti-cancer [18,19,20], inhibitors of diabetes controlling enzymes [21,22], antimicrobial [23], anti-inflammatory [22,24], anti-coagulant [22], skin brightening [25], etc.

Recently, NADES were described for the extraction of phlorotannins [26], ascorbic acid, and fucoxanthin from *F. vesiculosus* [9]. However, in the available literature, we have not found information regarding the ability of NADES for trace elements co-extraction from the brown seaweed *F. vesiculosus.*

The aim of this study was to investigate the ability of common NADES for several trace elements co-extraction from *F. vesiculosus* by different extraction techniques.

## 2. Results and Discussion

NADES can be formed by mixing a non-toxic quaternary ammonium salt, such as choline chloride (ChCl) and low toxicity hydrogen bond donor (HBD) compounds, such as urea, polyols, sugar, and organic acids [27]. The eutectic point in NADES is due to hydrogen bonding between the chloride of choline chloride and the protons in HBDs. Choline chloride is generally recognized as safe (GRAS) and is known for its nutritional benefits, for example, as a supplement to lower cholesterol levels. Several inexpensive, non-toxic, non-flammable, and biodegradable natural HBDs are available. Glucose, known as grape sugar, is a monosaccharide found in plants. Malic acid as dicarboxylic acid can be isolated from many fruits, such as apples, grapes, and vegetables. Lactic acid is a natural carboxylic acid present in milk and vegetables, which can be easily produced from carbohydrates by fermentation. These compounds are selected as HBDs in the proposed study.

### 2.1. NADES Composition Effect 

The total content of elements in dry seaweed *F. vesiculosus,* which is determined by inductively coupled plasma atomic emission spectroscopy (ICP-OES), was 20,553 ± 143 mg/kg DW (RSD 0.70%). The amount of individual elements was 112.53 ± 1.86 mg/kg DW (RSD 1.65%) for Al, 21.52 ± 0.75 mg/kg DW (RSD 3.48%) for As, 8.38 ± 0.06 mg/kg DW (RSD 0.76%) for Ba, 11967.74 ± 33.68 mg/kg DW (RSD 0.28%) for Ca, 0.65 ± 0.01 mg/kg DW (RSD 1.54%) for Co, 3.18 ± 0.07 mg/kg DW (RSD 2.27%) for Cr, 2.03 ± 0.08 mg/kg DW (RSD 4.10%) for Cu, 245.54 ± 0.84 mg/kg DW (RSD 0.34%) for Fe, 7306.45 ± 101.01 mg/kg DW (RSD 1.38%) for Mg, 71.35 ± 0.30 mg/kg DW (RSD 0.42%) for Mn, 767.74 ± 5.01 mg/kg DW (RSD 0.65%) for Sr, and 45.64 ± 0.09 mg/kg DW (RSD 0.19%) for Zn. Seaweeds contain relatively high amounts of Al, Ca, Mg, Fe, and Sr, while the concentrations of As (total), Ba, Mn, and Zn were relatively less. The lowest concentration was obtained for Co, Cu, and Cr. Some other elements, such as Bi, Cd, Ni, and Pb were not detected in *F. vesiculosus* (concentration was <LOQ). For further consideration, only elements with a concentration higher than LOQ were selected (Al, As, Ba, Ca, Co, Cr, Cu, Fe, Mg, Mn, Sr, and Zn). The results are in agreement with the earlier studies reported by [28], but it should be noted that the difference in the content of metals in seaweeds depends on the place of collection, the reproductive phase, and many other factors.

Polyphenols are one of the groups of biologically active substances of *F. vesiculosus,* for the extraction of 70% acetone, water, and NADES, which are most often used [21,26,29]. The trace elements could be co-extracted with polyphenols from seaweeds. A solid–liquid extraction (SLE) of *F. vesiculosus* was performed using three common acid-based NADES (Table 1), 70% acetone, and water. The most common NADES used for the extraction of polyphenols as well as lipophilic and hydrophilic compounds [9,26] from *F. vesiculosus* were selected. Similar solvents have been used for the extraction of polyphenolic compounds by other authors, as well [1,2,5,6,30]. The application of acid-based NADES resulted in creasing of the yields of active compounds; acid-based NADES are able to react with some metals and dissolve their oxides [31]. Tsvetov and Drogobuzhskaya (2021) showed that acidic NADES have good recovery of metals from *Empetrum nigrum* [32]. The ease of synthesis, as well as the availability and biodegradability of the components develop these acid-based versatile NADES [33].

**Figure 1:**
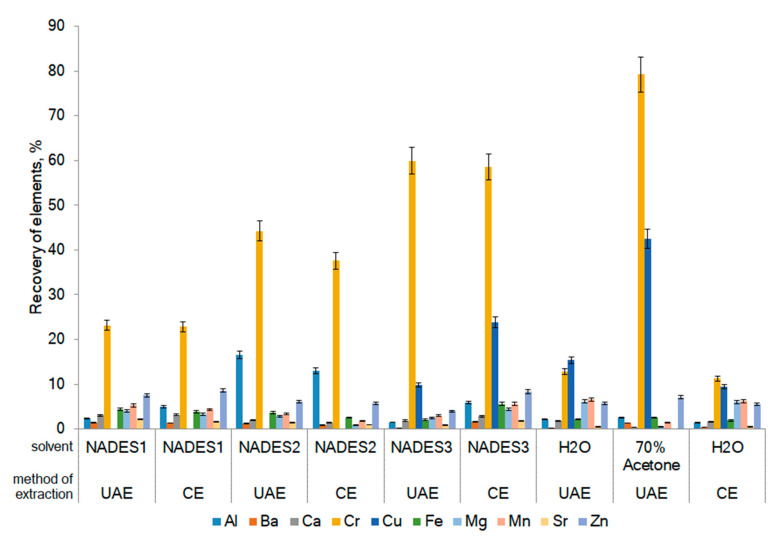
Recovery of trace elements from *F. vesiculosus* using NADES and conventional solvents (water and 70% acetone). UAE: Ultrasound-assisted extraction; CE: Conventional extraction.

NADES (Table 1) were tested under constant extraction conditions (solvent ratio of 1:10, an extraction time of 60 min, an extraction temperature of 60 °C), and a stirring speed of 700 rpm in the case of conventional extraction (CE). Significant differences in the co-extraction of trace metals with the tested NADES were reflected by the profiles (Table 2) and the calculated recovery of elements (Figure 1). It is interesting to note that all of the used NADES did not recover As and Co (concentration < LOQ, Table 1); the low recovery of elements, such as Ba, Ca, Fe, Mg, Mn, Sr, and Zn (recovery < 9%), were found in NADES extracts. The recovery of Al and Cr was relatively high and considerably varied (from 1.5 to 59.9%).

The recovery of Cr and Cu using extraction with 70% acetone is greater than the recovery of the same elements using water as conventional solvents (6.6 times and 3.4-folds for Cr and Cu, respectively). The recovery of Cr with all of the tested NADES was higher in 1.9–5-folds when compared with water, but less by the extraction with 70% acetone in 1.3–3.5-folds. Relatively high recovery (about 10%) was noted for the elements, such as Fe, Mg, Mn, and Zn, when using water as a solvent (Figure 1). It is interesting to note that arsenic and cobalt were not extracted by the NADES used in this work or conventional solvents (water and 70% acetone). The results revealed that the extracted amounts of main elements were in general higher for conventional solvents compared with NADES. The method of extraction does not statistically significantly affect the extractability of trace elements.

Several studies have revealed the effect of water addition on the physicochemical properties and supramolecular structure of deep eutectic solvents (DES) [34]. As previously reported, dilution with water above 50% (*v/v*) significantly weakened the intermolecular interactions of NADES based on choline chloride, with loss of the physicochemical properties of the eutectic solvent [5]. Water, as part of the supramolecular structure, can be strongly retained in the solvent and cannot be evaporated [35]. Moreover, the dilution method of hydrogen bond donor and hydrogen bond acceptor clusters in water with the increasing water absorption were previously shown, while hydrogen bonding is maintained upon dilution [36]. The addition of water to NADES may lead to weakening of the hydrogen–bonding interaction between the components of the solvents [37,38]. For this reason, the effect of the amount of water on the composition of NADES was investigated. The percentage of water in NADES advantageously reduces their viscosity, which facilitates the transfer of the analytes during extraction. The water content in NADES from 0 to 20% was not tested due to the obtained high extractants viscosity, which can disrupt the mixing process with the seaweed sample and filtration. Therefore, the addition of a range of water contents, from 20 to 80%, was investigated.

**Figure 2:**
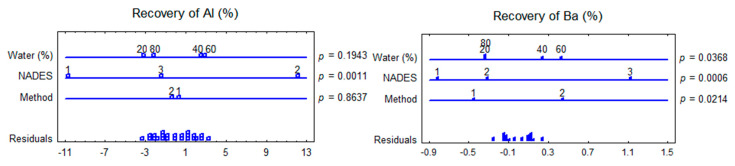

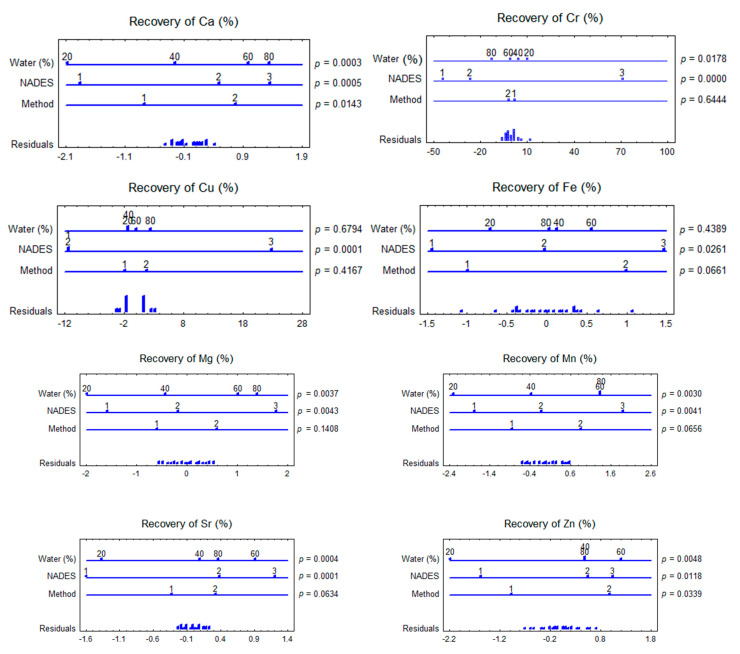
Multifactorial ANOVA plots showing the impact of NADES composition, water content in NADES, and extraction method on trace elements recovery from *F. vesiculosus. p*-values lower than 0.05 indicated a statistically significant effect at a confidence level of 95%. Method: UAE (1), SE (2); NADES:NADES1 (1), NADES2 (2), NADES3 (3) (Table 1).

The impact of NADES composition, water content in NADES, and the extraction method on the co-extraction of trace elements from *F. vesiculosus* were evaluated using multifactorial ANOVA (Figure 2). The multifactorial ANOVA test showed significant differences in the recovery of elements from *F. vesiculosus* when different NADES with variable water contents and different extraction methods were used. The method of extraction had not shown a statistically significant effect at a confidence level of 95% on the co-extraction of all elements (excluding Ba and Ca). In contrast, the water content in NADES was significantly affected on the recovery of Ba, Ca, Mg, Mn, Sr, and Zn. NADES1 provided the lowest contents of all elements, and the highest extracted amounts were obtained employing water contents of 60–80%. 

**Figure 3:**
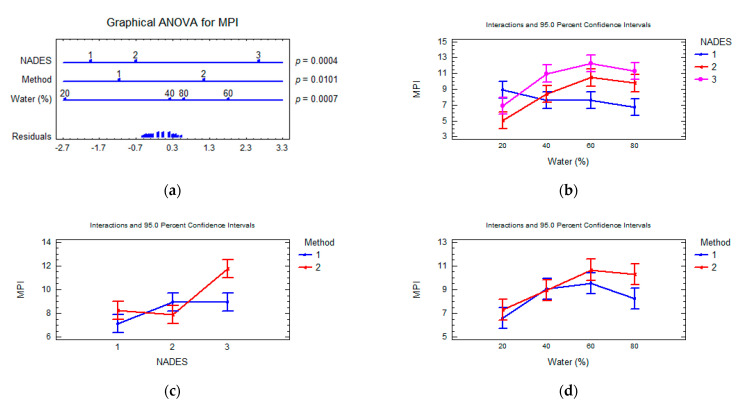
Multifactorial ANOVA plots. Influence of extraction parameters and composition of NADES on the metal pollution index. (**a**) Factors evaluated and *p*-values obtained; (**b**); interaction plot of content water in NADES and composition of NADES; (**c**) interaction plot of composition of NADES and extraction method; (**d**) interaction plot of extraction method and content water in NADES. Multifactorial ANOVA plots showing the influence. *p*-values lower than 0.05 indicated a statistically significant effect at a confidence level of 95%. Method: UAE (1), SE (2); NADES:NADES1 (1), NADES2 (2), NADES3 (3) (q.v. Table 1).

The metal pollution indexes (MPI) were calculated using the mean concentration of all tested elements. The MPI calculated with mean values of all sampling NADES extracts of *F. vesiculosus* turned out to be 9.8 ± 2.8 and varied from 5.1 (NADES3, UAE, 20% water) to 14.9 (NADES1, UAE, 60% water). The multifactorial ANOVA test showed a significant effect in total co-extraction of MPI metals (*p*-value < 0.05) for all principal factors studied (Figure 3). The composition of NADES is considered as the most important (the degree of the factor influence 23.3%, *p*-value 0.0004) and content of water in NADES composition (the degree of the factor influence 24.8%, *p*-value 0.0007). The interaction effect between the NADES composition and water content significantly affects the MPI value (the degree of the factor influence 31.6%, *p*-value 0.0015).

In recent years, NADES has been widely investigated for the extraction of food and medicinal plants as well as seaweeds. However, the ability of NADES for trace elements co-extraction from natural sources is not well investigated. We have found only a few publications in which the recovery of some trace elements by DES was reported.

The composition of NADES plays an important role for the extractability of different elements. Osowska and Ruzik [14] found that when dry young barley grass (*Hordeum vulgare* L.) are extracted with various NADES (1:20) using the vortexing method for 30 min, some metals are extracted and in a wide range. The highest extraction efficiency was for Zn and Mo (on average 45–50%), the extraction efficiency for Mn and Cu varied less: for Mn from 10 to 30%, for Cu from <LOQ to 30%. The efficiency of Cu extraction that is less than LOQ was found when using choline chloride as hydrogen bond acceptors of NADES and HBD compounds with the addition of water, such as sugars (glucose and fructose) (1:1:10 mol. ratio), glycerol (1:2:8 mol. ratio), and ethylene glycol and betaine (1:2:1:8 mol. ratio). However, in the case of using NADES citric acid and fructose (1:1:10), the efficiency of copper extraction increased up to 24%, and when using ChCl:ethylene glycol:water (1:4:8 mol. ratio) up to 30%. In our current study, we also observed a wide range of concentrations for Cu: Recovery varied from <LOQ to 31% (Figure 2). Lactic-based NADES does not extract Cu from seaweed. The recovery of Mn from *F. vesiculosus* by NADES used in the current study was considerably weaker from <LOQ to 3% (for malic acid based NADES).

The increase of water content in NADES (β-alanine:citric acid, 1:1) resulted in the rise of extraction efficiency of Mn, Cu, Mo, and Zn from *H. vulgare* grass [39]. These data are in line with our results. The increase of water in all of the tested NADES led to the increase of all the elements of recovery (Figure 2). The addition of water to NADES led to a decrease in the viscosity and surface tension, which increased the mass transfer from algal cells to the extract [40].

The aerial part of *Empetrum nigrum* L. was extracted with DES containing choline chloride and malonic, malic or citric acid (all 1:1) or tartaric acid (2:1) with 30 wt% water using UAE (1:10 *m*/*v* ratio) at 45 °C for 3 h. The extract contained relatively high concentrations of some metals (Si, Zn, Cu, Ni, and Mn) [32]. Apparently, a significant extraction of elements was affected by the long exposure time with ultrasound and temperature.

The recovery of several trace elements from red seaweed *P. haitanensis* was studied after the application of 28 NADES in a ratio of 1:20. It was found that when using NADES (malic acid:ChCl:water 1:1:2), the recovery of Cu was approx. 69%, recovery of As was approx. 21%, and recovery of Cr was approx. 24% [15], while in our experiment using NADES3 (malic acid:ChCl 1:1) on average, the Cu recovery was 9–30%, Cr recovery was 10–66%, and As recovery was less than LOQ. We believe that the lower content of elements in our experiment may be due to the specificity of seaweed and the absence of water in the composition of NADES.

We have found one report, in which the recovery of some trace elements by NADES (ChCl:glycerol, 1:2) with 50, 30, and 10 wt% water using UAE (*m*/*v* 1:10) at 50 °C for 3 h from the inflorescent of *Koenigia weyrichii* (syn. *Polygonum weyrichii* F. Schmidt) was investigated [41]. Analysis of elements in extracts by the inductively coupled plasma mass spectrometry technique showed that most of the elements have a positive correlation between the recovery and water content in DES. Toxic elements have a relatively low recovery, with the exception of Cd, which is practically 100% extracted. High recovery is typical for Zn.

NADES have demonstrated their usefulness in obtaining bioactive extracts from several sources, among them are agricultural by-products [1,2,3,4,5,6,7,8,9,10,11,12,13,42]. For the first time, the results of the current study demonstrated that NADES extracts of *F. vesiculosus* contain a lower amount of trace metals than the extracts obtained with water and 70% acetone. This indicates a significant advantage for NADES compared with the other solvents.

### 2.2. Human Health Risk Assessments of NADES Extracts

The toxicity of extracts contaminated with heavy metals for humans is associated with their daily intake [43]. There are three main routes of entry of pollutants into the human body: (a) Inhalation through the mouth and nose, (b) absorption through the skin, and (c) direct ingestion. NADES have a negligible vapor pressure [44,45]. Therefore, the health risk from inhalation of NADES extracts has not been evaluated. NADES extracts are used for topical application. The USEPA model and their threshold values [1989] were used to assess the potential human health risks posed by heavy metal pollution [46]. The chronic daily intake (CDI) of all tested elements was calculated according to the mean concentration of each metal in NADES extracts. The mean total daily intake of Al, Ba, Ca, Cr, Cu, Fe, Mg, Mn, Sr, and Zn was calculated as 2.20 × 10^−8^, 3.21 × 10^−10^, 9.19 × 10^−7^, 2.78 × 10^−9^, 3.33 × 10^−10^, 2.86 × 10^−8^, 6.68 × 10^−7^, 7.85 × 10^−9^, 3.35 × 10^−9^, and 8.93 × 10^−9^ mg/day, respectively. Daily intakes of all the metals are less than the daily dose risk estimators (Table 2). In the NADES extracts, mean values of CDI are decreased in the order: Mg > Ca > Fe > Sr > Mn > Al > Zn > Cr > Cu > Ba.

**Figure 4:**
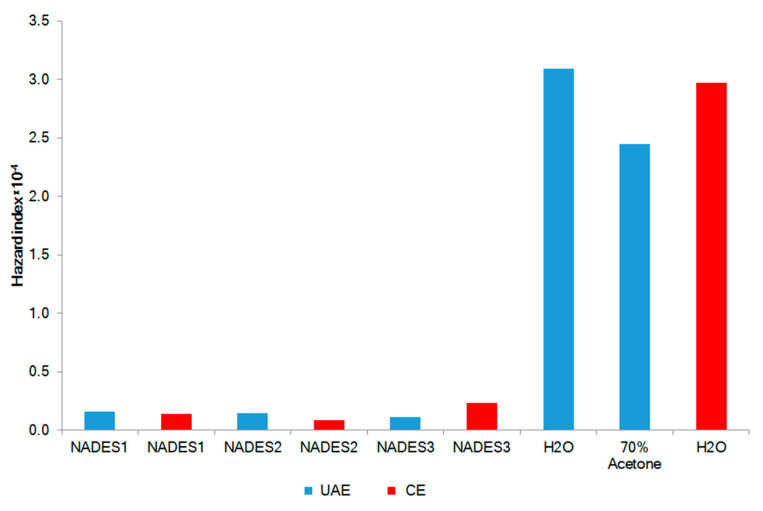
Non-carcinogenic risk (HI) of *F. vesiculosus* extract. UAE: Ultrasound-assisted extraction; CE: Conventional extraction.

The hazard quotient (HQ) is used for the health risks assessment of contaminated medicinal products for topical application. HQ is the ratio of a determined dose of pollutant to a reference dose level. The HQ >1 shows likely the negative impact of the product [47]. The HQ levels of the studied metals Al, Ba, Ca, Cr, Cu, Fe, Mg, Mn, Sr, and Zn were calculated as 2.19 × 10^−7^, 6.54 × 10^−8^, 1.15 × 10^−7^, 4.63 × 10^−6^, 2.78 × 10^−8^, 4.09 × 10^−8^, 4.77 × 10^−6^, 4.27 × 10^−6^, 2.79 × 10^−7^, and 1.49 × 10^−7^, respectively. The HQ levels for all the metals were considerably less than 1, showing no significant health risk via dermal adsorption of the studied NADES extracts. The combined non-carcinogenic effect of multiple elements is expressed by the hazard index (HI). The mean HI for NADES extracts from *F. vesiculosus* (HI = 1.46 × 10^−5^ ± 5.03 × 10^−6^) as well as from aqueous and 70% acetone extracts (HI= 2.83 × 10^−4^ ± 3.41 × 10^−5^) were less than 1 (Figure 4). This shows no carcinogenic risk after topical application.

Carcinogenic risk (CR) after topical application was calculated only for chromium using the dermal slope factor. CR between 10−6 and 10−4 is considered as acceptable [48]. We have found that the cancer risk of chromium in NADES extracts from *F. vesiculosus* (mean value CR = 5.55 × 10^−8^ ± 2.88 × 10^−8^) as well as in aqueous and 70% acetone extracts (mean value CR = 1.03 × 10^−6^ ± 1.15 × 10^−6^) was lower than the negligible range. This shows no carcinogenic risk from Cr consumption from dermal exposure of the NADES extracts from *F. vesiculosus*.

The calculated amount of several elements in a daily dose of NADES extracts of *F. vesiculosus* allowed us to understand the benefits/risks of daily consumption of seaweed extracts. The dose of one tablespoon (20 g) of seaweed NADES extracts was considered as the average daily consumption. In Table 3, we summarize the data at which the maximal concentration of a particular element was detected in the NADES extracts. Then, the maximal amount of elements consumed with 20 g of NADES extracts from *F. vesiculosus* was calculated and compared with the risk estimations for a 70-kg man [49,50,51,52] and nutritional requirements [52,53]. According to the data presented in Table 3, we can consider NADES extracts of *F. vesiculosus* as non-toxic and a valuable source of dietary elements that meet daily nutritional requirements.

## 3. Materials and Methods

### 3.1. Materials and Reagents

Arctic brown seaweed F. vesiculosus L. was collected from the littoral of the Barents Sea (Zelentskaya Bay, Murmansk region, Dalnie Zelentsy, Russia), identified by Dr. E. Obluchinskaya (voucher specimens 7.2021, E.D.O.) and deposited in the Collection of the Zoobentos Laboratory (Murmansk Marine Biology Institute, Murmansk, Russia). The seaweeds were carefully washed, cleaned from epiphytes, and dried at room temperature for 3 days to remove the surface moisture. Following its placement in a 50 °C vacuum oven for 1 to 2 days, the dried samples were pulverized using a Cyclotec mill (CT 293 Cyclotec, Foss, Hilleroed, Denmark) to pass through a screen with an aperture of 1.0 mm. Choline chloride was purchased from Acros Organics (Fair Lawn, NJ, USA), L-lactic acid and D(+)-glucose were from Panreac Química SLU (Barcelona, Spain), and DL-malic acid was from Sigma-Aldrich (St. Louis, MO, USA). 

### 3.2. Solvents Used

NADES were prepared by the heating method [54]. The ratios of components are presented in Table 1. The water was added to prepare NADES based on % weight. All of the other solvents were of analytical grade from local suppliers.

### 3.3. Extraction Procedures

Seaweed samples were mixed at a ratio of 1:10 (*w*/*v*) with one of the solvent. The ultrasound-assisted extraction was performed using the Branson MT-3510 ultrasonic bath (Branson Ultrasonics Corporation, Danbury, CT, USA) operated at 42 kHz, 130 W for 1 h. The maceration with magnetic stirring (1 h, 700 rpm) and heating at 60 °C were used for conventional extraction. Following the extraction, the samples were left at room temperature for 1 h and centrifuged. The supernatant was filtered with 0.45-μm syringe filter (Sigma-Aldrich, Bellefonte, PA, USA) and used for further analysis. All of the extraction procedures were performed in triplicate. 

### 3.4. Elements Analysis

A PerkinElmer^®^ Optima™ 8000 Model inductively coupled plasma optic emission spectrophotometer (ICP-OES) (PerkinElmer, Inc., Shelton, CT, USA) was used to quantify the metal ions in seaweed and NADES samples [55]. The instrument was optimized daily before the measurements and operated as recommended by the manufacturer. The instrumental parameters were plasma gas flow—10 L/min; auxiliary argon flow rate—0.2 L/min; nebulizer gas flow rate—0.7 L/min; plasma power—1300 W; and sample flow rate—1.5 mL/min. Microwave digestion unit Speedwave Entry Two (Berghof, Eningen unter Achalm, Germany) was used for the decomposition of plant samples before analyses by ICP-OES [56]. Analytical signals were measured as emission intensity values. All of the measurements were performed using argon gas to form the plasma. The wavelengths (nm) were Al 396.153; As 188.979; Ba 455.403; Bi 223.061; Ca 317.933; Cd 214.440; Co 238.892; Cr 267.716; Cu 327.393; Fe 238.204; Mg 279.077; Mn 257.610; Ni 231.604; Pb 220.353; Sr 407.771; Zn 213.857. 

Sample aliquots of approximately 500 mg were digested using 5 mL HNO_3_. Blank solutions were prepared by applying the same procedure and reagent solutions without the sample. The digestion program consisted of three steps: Room temperature to 150 °C in 5 min; 150–190 °C in 10 min; 190–75 °C in 15 min. Following the cooling to room temperature, the digested material was transferred to a 50 mL volumetric flask and the volume was set with ultrapure water. Analytical signals were measured as emission intensity values.

The yield of extraction (recovery, %) was calculated [14].

The total content of metals was obtained from three independent experiments and the mean value was calculated for each metal. Relative standard deviations (RSD) among the replicates of analysis of each sample were always lower than 5%. The limits of detection (LOD) and quantification (LOQ) are defined as the concentration corresponding to three and six times of the standard deviation (SD) of the blank, respectively, divided by the slope of the calibration curve. The LOD was calculated for standard deviations (SD) of six measurements for the blank. The LOQ of analyzed elements were as follows (mg/kg): Mn 0.59; Co 0.05; Cu 0.33; Zn 1.2; and Mo 0.12. The accuracy of the method was evaluated by adding a reference sample of Cu to a fucus sample, followed by extraction with NADES or a standard solvent. The recovery value of Cu was obtained from three independent experiments and the mean value was between 89 and 105%.

### 3.5. Human Health Risk Assessments

The chronic daily intake (CDI) was calculated by Equation (1) and the detailed explanation for all of the parameters are listed in [47]. The equation is adapted from the USEPA [46,57].

(1) $$\mathrm{CDI}=\frac{\mathrm{CS}\times \mathrm{SA}\times \mathrm{AF}\times \mathrm{ABS}\times \mathrm{EF}\times \mathrm{ED}\times \mathrm{CF}}{\mathrm{BW}\times \mathrm{AT}}$$

where CDI is the chronic daily intake through dermal absorption in mg/kg/day; CS is the average concentration of metal in extract of *F. vesiculosus* in mg/kg; SA is the exposed skin area in cm^2^; AF is the adherence factor in cm^2^/mg; ABS is the dermal absorption fraction; EF is the exposure frequency in day/year; ED is the exposure duration in years; CF depicts the units conversion factor in kg/mg; BW is the average body weight in kg; and AT is the averaging time in days for non-carcinogens.

The human health risk assessment was performed by calculating the hazard quotients (HQ, non-carcinogenic risk from individual metals) for metals and carcinogenic risk (CR) using Equations (2) and (3), respectively.

HQ = CDI_dermal_/RfD (2)

(3) $$CR=CDI_{dermal}\times SF\;$$

where RfD is the chronic reference dose of the toxicant in mg/kg/day; SF is the slope factor of hazardous substances in mg/kg/day obtained from USEPA by the integrated risk information system (IRIS) [58] database, which was 2 × 10^1^ mg/kg/day for CR.

To estimate the risk to human health through more than one heavy metal (HM), the hazard index (HI) has been developed [46]. The hazard index (HI) is the sum of the hazard quotients for all HQ, which was calculated using Equation (4) [59]:

(4) HI=∑HQ

The nutrimental importance of essential elements was assessed on the basis of nutritional requirements [52]. The health risk due to the toxic elements present in seaweeds was estimated using risk estimators [49,50,51,52,53].

The metal pollution index (MPI) [60,61] is a mathematical model that summarizes the composite influence of all the elements in the extract, and is calculated as the mean of values for the metals considered using Equation (5), as follows:

(5) $$\mathrm{MPI}={\left(M_{1}\times M_{2}\times \ldots \times M_{n}\right)}^{1/n}$$

where M_n_ is the concentration of the metal n in the sample in mg/kg.

### 3.6. Statistical Analysis 

Data were statistically analyzed using the multifactorial analysis of variance (ANOVA) to determine the effect of independent variables (extraction method and solvent, and content water in solvent) on the trace elements co-extraction from *F. vesiculosus.* All of the statistical analyses were performed with STATGRAPHICS Centurion XV (StatPoint Technologies Inc., Warrenton, VA, USA). Data are expressed as mean ± standard deviation (±SD), and the error bars in figures indicate the standard deviation. 

## 4. Conclusions

NADES are used for the extraction of different groups of biologically active compounds from natural sources. However, little is known regarding the ability of NADES for trace elements co-extraction. To fulfill this gap, we have compared the impact of the extraction method of NADES, water, and 70% acetone on the trace elements co-extraction from *F. vesiculosus.* The results revealed that the extracted amounts were in general higher for conventional solvents compared with NADES. All of the tested NADES did not recover As and Co (concentration <LOQ). Moreover, all of the tested NADES provided a low recovery of elements, such as Al, Ba, Ca, Fe, Mg, Mn, Sr, and Zn (recovery <9%). The method of extraction had not shown a statistically significant effect on all elements co-extraction (excluding Ba and Ca). In contrast, the water content in NADES was significantly affected on the recovery of Ba, Ca, Mg, Mn, Sr, and Zn. The recovery of Cr and Cu showed analogous behaviors, NADES1 provided the lowest contents of all elements, and the highest extracted amounts were obtained employing water contents of 60–80%.

The toxicity of extracts contaminated with trace metals for humans is related to their daily intake. Daily intakes of all the elements contained in NADES extracts are less than the daily dose risk estimators. The hazard quotients, hazard indexes, and carcinogenic risk calculated for all trace elements and their combination were considerably less than 1. This evidences no health risk, and carcinogenic risk after topical application of all studied NADES extracts from *F. vesiculosus.*

For the first time, the results of the current study demonstrated that the NADES extracts of *F. vesiculosus* contain a lower amount of trace metals and are safer than the extracts obtained with water and 70% acetone. This indicates a significant advantage for NADES compared with the other solvents. Additionally, according to the calculated daily consumption of elements with 20 g of extracts, we can consider the NADES extracts of *F. vesiculosus* as not only non-toxic, but also a valuable source of dietary elements that meet daily nutritional requirements.

## Figures and Tables

**Table 1:** Natural deep eutectic solvents (NADES) used for extraction.

NADES Code	Components	Molar Ratio	Appearance
NADES1	Lactic acid:glucose:H_2_O	5:3:1	Transparent liquid
NADES2	Lactic acid:ChCl	3:1	Viscous transparent colorless liquid
NADES3	Malic acid:ChCl	1:1	Viscous transparent colorless liquid

**Table 2:** The concentrations of elements (mg/kg) in samples of *Fucus vesiculosus* extracts (mean ± SD, *n* = 3).

Elements	LOQ	UAE					CE			
		NADES1	NADES2	NADES3	H20	70% Acetone	NADES1	NADES2	NADES3	H20
Al	1.6	3.15 ± 0.06	17.8 ± 2.9	2.12 ± 0.19	48.5 ± 0.6	56.5 ± 1.2	6.50 ± 0.10	14.08 ± 0.21	8.34 ± 0.16	33.4 ± 0.6
As	6.3	nd	nd	nd	nd	nd	nd	nd	nd	nd
Ba	0.016	0.15 ± 0	0.13 ± 0.01	0.030 ± 0.001	0.43 ± 0.01	2.25 ± 0.03	0.14 ± 0.00	0.093 ± 0.002	0.17 ± 0.00	0.55 ± 0.01
Ca	1.9	454 ± 2	304 ± 14	282 ± 4	4173 ± 40	918 ± 8	471 ± 3	224 ± 2	429 ± 4	4056 ± 38
Co	0.12	nd	nd	nd	nd	nd	nd	nd	nd	nd
Cr	0.13	0.76 ± 0.04	1.36 ± 0.02	2.28 ± 0.06	8.19 ± 0.16	50.4 ± 1.4	0.75 ± 0.05	1.15 ± 0.04	2.33 ± 0.04	7.19 ± 0.07
Cu	0.17	nd	nd	0.25 ± 0.02	6.24 ± 0.10	17.3 ± 0.7	nd	nd	0.60 ± 0.02	3.81 ± 0.11
Fe	0.098	13.6 ± 0.1	11.2 ± 0.1	6.30 ± 0.11	107 ± 1	124 ± 1	11.9 ± 0.1	7.67 ± 0.09	17.1 ± 0.1	93.9 ± 1.1
Mg	1.7	366 ± 3	263 ± 3	228 ± 4	8974 ± 74	723 ± 16	300 ± 4	80 ± 1	402 ± 5	8856 ± 68
Mn	0.058	4.71 ± 0.03	3.02 ± 0.02	2.64 ± 0.03	94.0 ± 0.9	20.9 ± 0.3	3.86 ± 0.04	1.53 ± 0.02	5.01 ± 0.04	89.2 ± 0.7
Sr	0.026	20.8 ± 0.2	13.3 ± 0.6	8.73 ± 0.11	77.1 ± 0.6	14.5 ± 0.1	15.8 ± 0.2	9.17 ± 0.07	17.6 ± 0.2	76.8 ± 0.7
Zn	0.17	4.26 ± 0.05	3.40 ± 0.02	2.25 ± 0.05	52.8 ± 0.5	64.8 ± 1.1	4.85 ± 0.04	3.15 ± 0.04	4.77 ± 0.08	49.8 ± 0.5

nd < LOQ. UAE: Ultrasound-assisted extraction; CE: Conventional extraction.

**Table 3:** Element maximum concentration (mg/kg), its daily dose (mg/day) in NADES extracts from *F. vesiculosus*, and comparison with daily dose risk estimators for a 70-kg man and nutritional requirements.

Element	NADES	Water Content, %	Maximum Concentration	Daily Dose for 20 g Consumption	Daily Dose from Risk Estimators	Daily Nutritional Requirements
Al	2	60	29.89	0.60	10 ^1^	
Ba	3	60	0.37	0.19		0.75 ^5^
Ca	3	80	1140	23	2500 ^2^	1000 ^3^
Cr	3	40	2.68	0.05	30 ^5^	0.05 ^5^
Cu	3	80	0.016	0.05	5 ^2,5^	0.9 ^4^/1.0 ^5^
Fe	3	60	18.82	0.38	45 ^5^	10 ^3,5^
Mg	3	80	692	14	800 ^5^	400 ^5^
Mn	3	80	8.04	0.16	11 ^5^	2.7 ^3^/2.0 ^5^
Sr	3	60	47.89	0.96	11 ^5^	1.9 ^5^
Zn	3	60	6.41	0.13	25 ^2^	12 ^3,5^

^1^ PTWI: Provisional tolerable weekly intake; ^2^ UL: Tolerable upper intake level; ^3^ PRI: Population reference intake; ^4^ AI: Adequate intake; ^5^ [53].

## Data Availability

The data are available on request from the corresponding author.
